# Complete mitogenome of *Gymnocorymbus ternetzi* (Characiformes: Characidae: *Gymnocorymbus*) and phylogenetic implications

**DOI:** 10.1080/23802359.2021.2008844

**Published:** 2021-12-15

**Authors:** Qunyin Zhu, Site Luo, Shang Pan, Xiaohan Su, Ziming Liu, Jie Chen

**Affiliations:** a College of Ecology, Lishui University, Lishui, China; b School of Life Sciences, Xiamen University, Xiamen, China

**Keywords:** *Gymnocorymbus ternetz*, mitochondrial genome, phylogenetic relationship

## Abstract

*Gymnocorymbus ternetzi* belongs to the genus Gymnocorymbus in the family Characidae, and is mainly distributed in southern Brazil. Herein, we report the complete mitogenome of *G. ternetzi* using Illumina sequencing data. The mitogenome is 17,999 bp in length and contains 13 protein-coding genes, 22 transfer RNA genes, and 2 ribosomal RNA genes. Phylogenetic analysis of *G. ternetzi* and 18 related species within Characidae indicates that *G. ternetzi* clusters within the family Characidae. The data provide useful genetic information for future studies on the taxonomy, phylogeny, and evolution of Characidae species.

Characidae is among the most diverse families of Characiformes and one of the largest clades of fish globally, with a complex taxonomic background (Benine et al. 2015). The black widow tetra *Gymnocorymbus ternetzi* (Boulenger, 1895), belonging to the genus *Gymnocorymbus* within the family Characidae, is a peaceful shoaling tropical freshwater fish distributed in the rivers of southern Brazil (Priestley et al., 2006). It is an omnivorous fish that feeds on plants, insects, and crustaceans (Polaz et al. 2017). Because of its attractive appearance, undemanding maintenance, and ease of breeding, *G. ternetzi* is a popular freshwater ornamental fish (Frankel 2004; Uma and Chandran 2008). The evolutionary genetics of *G. ternetzi* are unknown. In this study, we report the complete mitochondrial genome of this species. The data will be a valuable resource for further studies on evolutionary genetics, species delimitation, and phylogenetic studies on *Gymnocorymbus*.

Samples were collected from Pudong New Area, Shanghai City, China (31°19′06.89″N, 121°67′90.75″E). The voucher specimen was deposited at the Lishui University (No. LSU-ZJ2021-03-01–LSU-ZJ2021-03-10 by Ziming Liu, liuziming@lsu.edu.cn). Genomic DNA was extracted using a TIANamp Genomic DNA Kit (TIANGEN, Beijing, China). The sequencing library was produced using the Illumina Truseq™ DNA Sample Preparation Kit (Illumina, San Diego, CA, USA) according to the manufacturer's recommendations. The prepared library was loaded on an Illumina Novaseq 6000 platform for paired-end 2 × 150 bp sequencing at Novogene (Beijing, China). Raw data were used to assemble the complete mitogenome genome using the GetOrganelle pipeline (Jin et al. 2020). Genome annotation was performed using Mitoz annotation module (Meng et al. 2019). The annotated genome sequence was deposited in GenBank under accession number MZ363625.

The circular mitogenome of *G. ternetzi* is 17,999 bp in length, with 29.92% A, 28.41% T, 15.37% G, and 26.30% C. The greater A + T content (58.33%) than the G + C content (41.67%) indicated a slight A + T bias in *G. ternetzi*. Further, 37 genes were predicted, including 13 protein-coding genes, 22 transfer RNAs, and 2 ribosomal RNA genes.

Phylogenetic analysis was performed using complete mitogenomes from 23 species of *Paralichthys olivaceus* and *Cynoglossus semilaevis* serving as outgroup taxa. The genomes were aligned with MAFFT v7.388 using default settings (Katoh and Standley 2013). Phylogenetic analysis was conducted based on maximum likelihood (ML) analyses implemented in IQ-TREE v2.1.2 with the GTR + F+R2 nucleotide substitution model selected by ModelFinder (Kalyaanamoorthy et al. 2017; Minh et al. 2020). Support for the inferred ML tree was inferred by bootstrapping with 1,000 replicates. The analysis showed that *G. ternetzi* was in a clade with *Gephyrocharax atracaudatus* (Figure 1).

**Figure 1.**
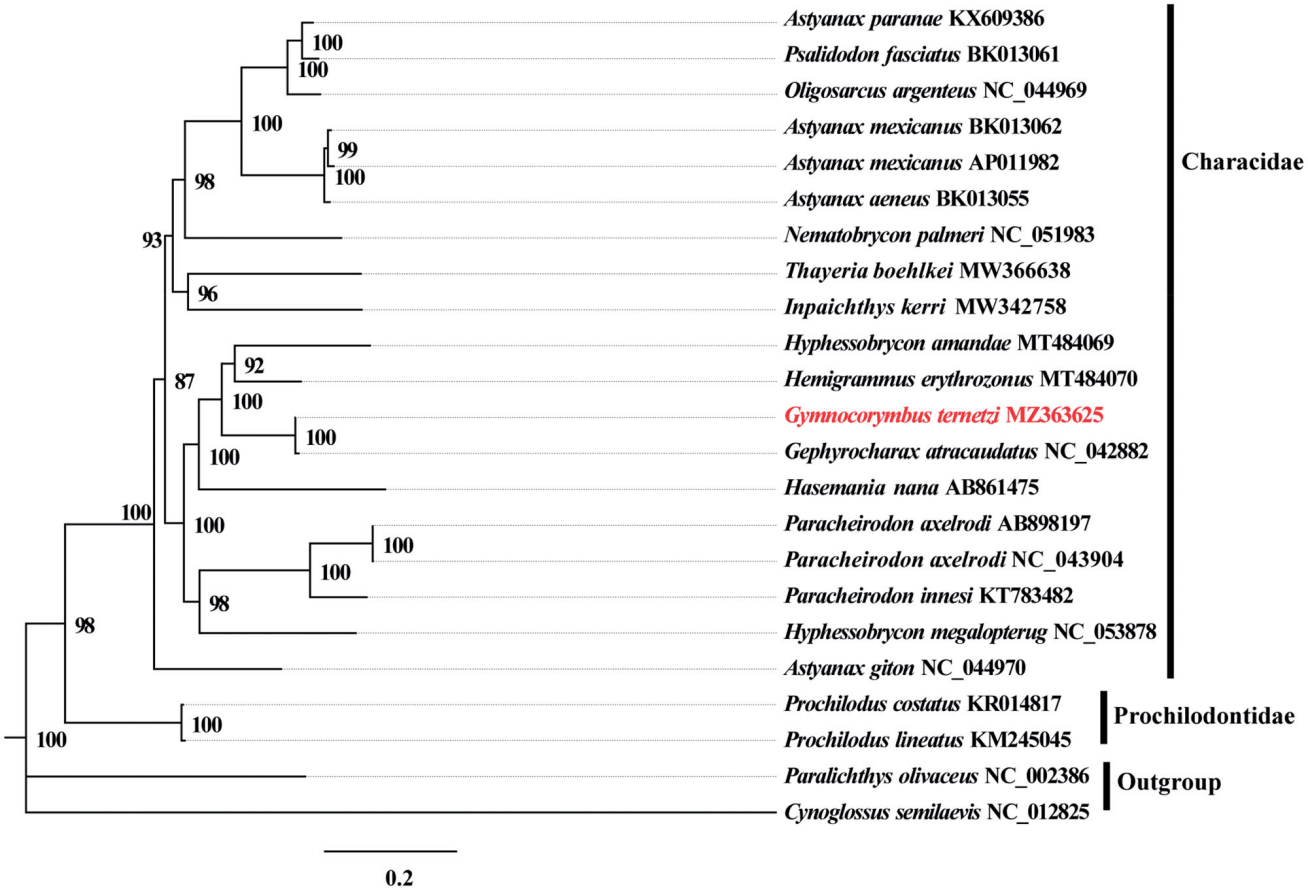
Maximum likelihood (ML) tree based on 19 mitogenome sequences of representative fish in Characidae as the ingroup and *Paralichthys olivaceus* and *Cynoglossus semilaevis* as the outgroup. Numbers on the nodes are bootstrap values based on 1,000 replicates. The *G. ternetzi* genome is marked in bold and red font.

This study provides important sequence information for species identification and phylogenetic relationships in the Characidae species.

## Data Availability

The genome sequence data that support the findings of this study are openly available in GenBank (https://www.ncbi.nlm.nih.gov/) under accession no MZ363625. The associated BioProject, SRA, and Bio-Sample numbers are PRJNA725085, SAMN18869625, and SRR14322678, respectively.
